# Local varieties of cassava: conservation, cultivation and use in Uganda

**DOI:** 10.1007/s10668-017-9997-6

**Published:** 2017-06-28

**Authors:** Grace Nakabonge, C. Samukoya, Y. Baguma

**Affiliations:** 1 National Crops Resources Research Institute (NaCRRI), P. O. Box 7084, Kampala, Uganda; 2 National Agricultural Research Organization (NARO), P.O. Box 295, Entebbe, Uganda; 3 College of Agriculture and Environmental Sciences, Makerere University, P.O. Box 7062, Kampala, Uganda

**Keywords:** On farm, Socio-cultural, Germplasm, Traditional farmer knowledge

## Abstract

The study explored the theory that on-farm conservation of cassava germplasm is influenced by farmers’ traditional and cultural preferences of particular varieties. Traditional knowledge practices that are used for on-farm conservation of cassava germplasm as well as cassava attributes for selection were assessed. The findings obtained from the study indicated that farmers use traditional knowledge to select and preserve cassava germplasm for future use. It was also clear that farmers have their preferences such as culinary attributes, storability in the ground, early maturity and cooking quality to mention but a few that influence the decisions taken to retain or abandon cultivation of varieties. Therefore, by planting varieties in multiples plots, replanting immediately after harvesting, sharing with others in the community and planting disease-free materials, farmers ensure that they preserve varieties of interest for decades. The information generated during this study could inform development policies tailored toward ensuring sustainable on-farm conservation of cassava genetic resources.

## 1 Introduction

Crop genetic resources are important in ensuring food security since they provide the raw materials needed for crop improvement (FAO 2010). Farmers over the years have cultivated a variety of crops on their farms that are adapted to particular needs and conditions (Tripp 1996). It is from the human selection practices that crop genetic resources have been preserved for many decades (Gwali et al. 2011). However, the introduction of improved varieties originating from crop improvement programs to combat emergence of diseases and pests as well as other environmental stresses are subjecting several crops genetic resources to genetic erosion (Peroni and Hanazaki 2002; Legg and Fauquet 2004; Alicai et al. 2007). It is important to support conservation of crop genetic resources by fully understanding the traditional knowledge and practices that influence their selection maintenance and conservation (Negi 2010; Parajuli and Das 2013; Wilder et al. 2016). It is important to be mindful of the notion that farmers may not preserve varieties for the sake of conservation but rather because they are adapted to particular needs and conditions that may change at any given time (Tripp 1996). Traditional farmer knowledge is not only important for its socio-cultural value but also for a number of reasons most of which are relevant to the conservation of crop biodiversity. While it has been the responsibility of genetic resource and research centers to recover germplasm before it is lost and to ensure its introduction into germplasm banks (FAO 2010), it is important that the role of local farmers in genetic resource conservation is recognized since they aid in ensuring food and agricultural diversity, valuable landscape, livelihoods and food security. Nevertheless, traditional livelihoods and indigenous plant varieties are increasingly endangered by largescale commercialization of agriculture, population dynamics, land-use/cover changes and the impacts of climate change (FAO 2009).

Cassava (*Manihot esculenta* Crantz) is one of the crops that have been conserved by farmers for decades, and it is thought to have been cultivated from 3000 to 7000 years ago in South America (Ng and Ng 2002). Cassava is an annual root crop that is widely thought to have originated from the Amazon basin (Nassar 2000). The crop was later introduced to West Africa from which it spread to other African nations (Hillocks 2002; Okogbenin et al. 2007). In Uganda Cassava was introduced between 1862 and 1875 by the Asian traders and spread to almost all parts of the country (Langlands 1972). Its spread and cultivation was due to its adaptability to a variety of agro-ecological conditions and its tolerance to drought (Nassar and Ortiz 2006; OECD 2014). Cassava is therefore regarded as a food security crop in Uganda and Africa as a whole (Balyejusa Kizito 2006). Currently, Cassava is grown in many parts of Uganda as one of the major food crops and is globally gaining economic importance for its starch utilization in food, feed and industry (Legg 1999; Jansson et al. 2009; Nuwamanya et al. 2009; Turyagyenda et al. 2012).

The duration of cassava cultivation in Uganda implies that it has evolved over time and has undergone both environmental and human selection on farmers’ fields. Cassava is consequently thought to be harboring important genes that will be of use in the future improvement of the crop (Turyagyenda et al. 2012). However, its genetic diversity is threatened by diseases most notably cassava mosaic and cassava brown streak diseases that have resulted into reduced productivity and loss of germplasm (Legg and Fauquet 2004; Alicai et al. 2007; Kawuki et al. 2016). The current global focus of breeding cassava varieties for industrial purposes will further threaten the cassava genetic resources as farmers will focus on growing cassava for commercial purposes (Jansson et al. 2009). For instance, in Teso region, the farmers are mainly growing NaSe_3_, the improved variety from cassava breeding program. It is difficult to find landraces like Ebwanatereka and Jaribu which were once common (Mr. Okaasai Opolot. personal communication February 10 2014). The loss of cassava genetic resources might compromise future breeding of the crop thus the need for support toward its ex situ and in situ conservation.

Previous studies on management and differentiation of local varieties by farmers in Uganda revealed that there could be variation in on-farm selection and cultivation of varieties influenced by cultural views (Balyejusa Kizito et al. 2006). For instance, some cultures do not cultivate bitter varieties because they do not consider them as food, whereas other cultures mostly in mid-northern Uganda and northwestern cultivate mostly bitter varieties that are considered to be tastier after processing than sweet varieties (Balyejusa Kizito et al. 2006, 2007). Bitterness is also a security measure as thieves cannot readily use/sell them. Therefore, farmers have perceptions about local varieties that need to be fully understood and integrated into future on-farm conservation policy that is acceptable and legitimate to the local communities. Understanding the socio-cultural factors that influence farmer decision making during the selection and retention of cassava varietal diversity is crucial for future improvement of the crop. The current study was aimed at assessing the farmer knowledge and practices that influence decision making in selection and retention of cassava local varieties in 6 agro-ecological zones of Uganda. The objectives were to document farmer varieties based on farmers’ knowledge, to determine the farmers’ preferences for cultivation, utilization and selection, and to assess conservation practices undertaken by farmers on the cassava varieties. Undertaking a study that will generate knowledge on conservation of food security crop like cassava is useful in the quest for attaining sustainable development goal 2 which focuses on ending hunger and all sorts of malnutrition by 2030. Furthermore, the study responds to agenda 21 of the earth summit that calls for programs and policies for strengthening biodiversity conservation for sustainable economic development and environmental protection.

## 2 Methods

### 2.1 Study area

The study was conducted in Uganda which is located in East Africa and lies astride the Equator, between latitudes 4°12′N and 1°29′S and longitudes 29°340′W and 35°00′E. Temperatures are in the range of 15°–30 °C. The country can be suitably divided into seven broad agro-ecological zones which have similar economic and social backgrounds, and in which ecological conditions (soil types, topography, rainfall), farming systems and practices are fairly homogeneous (UBOS 2010). Six agro-ecological zones southwest, mid-west, mid-north, eastern, central and northwestern Uganda were surveyed. In eastern agroecological zone, districts of Kaberamaido, Soroti, Amuria, Serere, Mbale, Manafwa, Busia and Iganga were surveyed. Southwestern districts of Rukungiri, Bushenyi, Buhinga and Ntungamo were surveyed. Mid-western districts surveyed include Kasese, Hoima, Kibaale, Kabalore, Buliisa, Kiryandongo, Kyenjojo, Kyegegwa and Kiryandongo. In northwestern Uganda, districts of Koboko, Nebbi, Arua were surveyed, whereas in central agro-ecological zone, districts of Kalangala, Butambala. Masaka, Kayunga, Luwero, Mukono, Rakai, Nakaseke, Bukomansimbi and Kalungu were surveyed. Mid-northern districts of Gulu, Lira and Apac were surveyed. The study focused on collecting cassava local varieties and the associated traditional knowledge for in vitro conservation. In this paper, a “local variety” is a cassava landrace identified by farmers under a single ethnic name which had been grown in the area for at least 20 years. The data collection was conducted between 2013 and 2016.

### 2.2 Sampling strategy

**Fig. 1:**
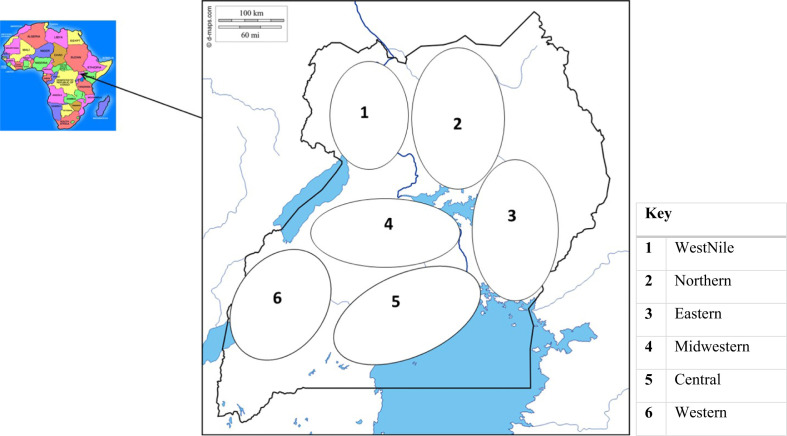
Location of the 6 agro-ecological zones of Uganda where the study was conducted

Districts from each of the 6 agro-ecological zones (Fig. 1) were selected purposively based on their track record in cassava growing. Sub-counties and households were purposively sampled using snowball method where prior information was obtained from extension workers at the districts that they possess cassava local varieties in their gardens. From each district, 4 sub-counties were further sampled. Households within sub-counties were sampled based on information from key informants or from the previous household about the presence of cassava local varieties. A total of 384 respondents were interviewed using a questionnaire across all the selected regions. Variables investigated included farmer preferences for cassava cultivation, utilization and cassava conservation practices as well as cassava local varieties cultivated by farmers. Varieties were recorded as named by farmers in their native languages of the different ethnic groups in the different sites selected.

### 2.3 Data analysis

**Fig. 2:**
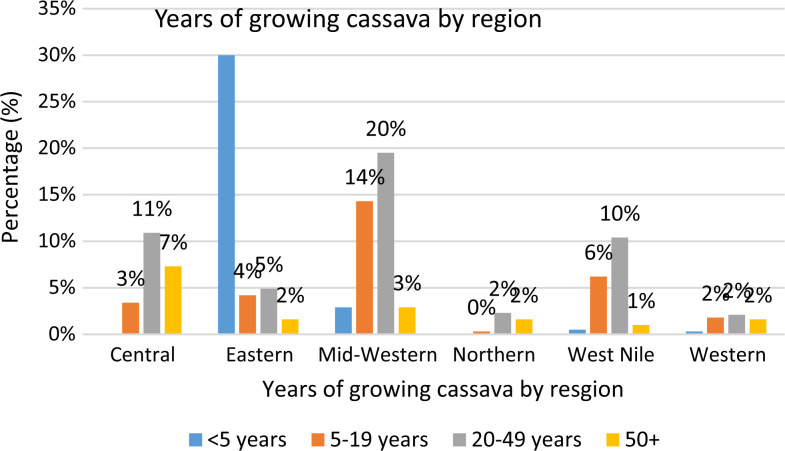
Duration of cassava cultivation in the study areas categorized into <5, 5–19, 20–49 and 50 years

Data were coded in Microsoft Excel and analyzed using the Statistical Package for Social Scientists (SPSS) version 24.0 (2016 release, _IBM Corp., Chicago, IL, USA) using descriptive statistics. Multiple response data for conservation practices and production of cassava local varieties were grouped together using the multiple response command of SPSS. The descriptive words provided for each use were categorized and counted through the multiple responses. Data were presented as frequencies in the households sampled, percentages and cross-tabs. Cassava local varieties were also listed using the ethnic names provided by farmers (Fig. 2).

## 3 Results

### 3.1 Duration of cassava cultivation

The information generated during this study was obtained from farmers who had been cultivating cassava for a period of not less than 5–50 years and more. Mid-western Uganda and central Uganda recorded the longest period of cassava cultivation in the study area with central Uganda having the highest number of farmers who reported having been growing cassava for the last 50 years and more. The mean age for respondents was 47 male and 46 female.

### 3.2 Production dynamics of cassava local varieties

#### 3.2.1 Acreage under cassava cultivation

In the current study, 60% of all households interviewed grow cassava on land between 1 and 4.9 acres followed by 32.7% that grow cassava on <1 acre, 4.9% on 5–9.9 acres and 1.8% on 10 or more acres. In mid-western, central, eastern, northwest and mid-north Uganda, majority of farmers grow cassava on 1–4.9 acres, at 19.7, 16.1, 7.5, 11.4 and 3.4%, respectively (Table 1).

**Table 1:** Acreage of cassava cultivation in central, eastern, mid-west, mid-north, northwest and west Uganda

Region	<1 acre	(%)	1–4.9 (%)	5–9.9 (%)	10+ (%)	Total (%)
*Area of land under cassava by region*						
Central	5.5		16.1			21.6
Eastern	2.6		7.5	0.5	0.3	10.9
Mid-west	15.3		19.7	2.9	1.6	39.5
Mid-north	0.8		3.4			4.2
Northwest	5.2		11.4	1.6		18.2
Southwest	3.4		2.3			5.7
Total	32.7		60.5	4.9	1.8	100.0

#### 3.2.1.1 Number of varieties cultivated by farmers

The current study indicated that farmers grow up to a maximum of 14 varieties on their farms. However, most farmers grow between 1 and 4 local varieties (Table 2). The highest number of varieties grown on farm was reported from northwestern and mid-western Uganda.

**Table 2:** Number of cassava varieties cultivated per household in the six regions based on farmer responses

Region	Number of varieties									Total
	1 (%)	2 (%)	3 (%)	4 (%)	5 (%)	6 (%)	7 (%)	8 (%)	14 (%)	
Central	11.2	4.4	2.1	2.1	0.0	0.0	1.8	0.0	0.0	21.6
Eastern	3.1	4.5	1.6	0.5	0.3	1.3	0.0	0.0	0.0	11.3
Mid-west	6.8	5.2	10.2	6.5	5.2	1.6	2.9	1.3	0.0	39.6
Mid-north	1.8	1.6	0.8	0.0	0.0	0.0	0.0	0.0	0.0	4.2
Northwest	0.8	1.8	1.6	3.9	4.2	1.3	1.0	0.0	3.6	18.2
West	3.9	1.6	0.0	0.0	0.0	0.0	0.0	0.0	0.0	5.5
Total	27.6	18.8	16.1	13.0	9.6	4.2	5.7	1.3	3.6	100.0

### 3.3 Local varieties of cassava in the different agro-ecological zones of Uganda

A total of 217 local varieties were reported by farmers and stakes collected for conservation at the National crops resources research institute (NaCRRI), Namulonge, Uganda. Of these, 49 were collected from northwest, 89 from mid-west, 33 from central, 11 from mid-north, 15 from southwest and 20 from eastern Uganda. Nyaraboke, Karangwa, Kabiriti and Kirimumpale were the common varieties from mid-western, whereas Gbasumenge, Abiriya, Mingoro and Sanje were common in northwest. Njule and Kwatamumpale were common in central Uganda. Magana and Ofumbachai were the common varieties in eastern, whereas Bao, Ogwok and Icilcil were common in mid-northern Uganda. Bukalasa was common across all agro-ecological zones surveyed (Table 1). Generally varieties were named based on place of origin (northwest 9, mid-west 14, central 8, southwest 1, mid-north 1), maturity period (northwest 3, mid-west 3, central 1, mid-north 1), taste (northwest 3, mid-west 8, central 3, southwest 2, eastern 2), morphology (northwest 4, mid-west 9, central 3, southwest 3, mid-north 3), ease to cook (northwest 1, mid-west 5, eastern 3), yield (northwest 1, mid-west 6, central 1, southwest 2, eastern 1), disease susceptibility and tolerance (mid-west 2) marketability (central 2, eastern 1), resilience (southwest 1). However, for some varieties farmers did not know the meaning of the names given (Table 3).

**Table 3:** Cassava varieties collected as named by farmers in the 6 agro-ecological zones

Area of origin/source	Northwest			
	Variety	Language	Meaning	Frequency
*(a) Categorization attribute*				
	Nyarukecha	Alur	From Okecha (name of person who introduced variety to the area)	3
	Drua	Lugbara	Many tubers	3
	Mingoro	Lugbara	Originated from Congo through the Mingoro Clan	7
	Nyamatya	Alur	Introduced to community by Matya	2
	Ochok-Ola	Lugbara	Mainly grown by Ochok tribe	3
	Abdu		Introduced to community by Abdu	3
	Bukalasa		From a place called Bukalasa	3
	Ariwara	Unknown from DRC	From a place called Ariwara in Congo	4
	Aliba gbanda	Lugbara	Gbanda meaning cassava; Aliba refers to name of person who introduced variety	2
Maturity period				
	Abiria	Lugbara	One that saves you from Hunger (because its early maturing)	7
	Godiri	Lugbara	Hard as tire (the variety is as hard as a tire and takes long to cook)	3
	Nyaruchanda	Alur	Something that wastes time (because variety takes long to mature)	1
Taste				
	Kali	Swahiri	Bitter	3
	Mabulu	Madi	Came to stay	3
	Sanje	Lugbara	You will see the benefits after planting (because its early maturing)	6
Morphology				
	Palawu	Lugbara	One that produces many leaves	6
	Alia	Kakwa	Elongated but tasty	2
	Ogangara	Alur	Variety grows many branches	1
	Derea	Lugbara	Short (variety grows short stems/branches)	1
	Ocol	Alur	Dark (cassava has dark stems)	
Ease to cook				
	Aluthumoni	Kakwa	In-law wait (in-law was told to wait for food as it cooks fast)	3
Yield				
	Gilagila	Kakwa	Help (variety needs a lot of management to yield)	1
Unknown attribute	Angaruba	Alur		1
	Gbasumenge	Lugbara		13
	Aliba gbanda	Lugbara		2
	Nyapamitu	Alur		4
	Amua	Alur		2
	Nyoeroli	Alur		2
	Joyo	Madi moyo		2
	Nyarudota	Alur		2
	Omoo	Lugbara		2
	Nyamuto	Alur		2
	Nyamukere	Alur		2
	Akulu	Kakwa		2
	Malokwa	Lugbara		2
	Sombili	Not known		1
	Thurungule	Not known		1

	Central			
	Variety name	Language	Meaning	Frequency
*(b) Categorization attribute*				
Area of origin/source	Bukalasa	Luganda	From a place called Bukalasa	7
	Kitobo	Luganda	Name of place where variety was obtained	2
	Naggalabi	Luganda	Name of place where variety was obtained	1
	Butenga	Luganda	Name of place where variety was obtained	1
	Kimaka	Luganda	Name of place where variety was obtained	1
	Nakanaabo	Luganda	Name of person who introduced variety to the area	1
	Nakyanzi	Luganda	-	3
	Lusula-	-	-	3
Morphology				
	Kalitunsi	Luganda	Eucalyptus (as tall as eucalyptus tree)	4
	Njule	Luganda	Opening (identified by the way leaves open/spread out)	5
	Dduka-obusolo	Luganda	Keep away from rodents (tubers enlarge a distance from stem, thus confuse animals that burrow around the stem looking for tubers to eat)	2
Marketability				
	Kwatamumpale	Luganda	Pocket (everybody who sees it gets money to buy) Marketable as well	8
	Kabwa	Luganda	Dog (meaning that variety can protect household from hunger in the same manner as dogs protect from enemies	2
Yield				
	Mpologoma	Luganda	Lion (cassava has large tubers)	3
Taste	Kitampunu	Runyankore	Kills pigs (very bitter)	3
	Kawogo	Luganda	Precious cassava variety (very tasty)	2
	Matooke	Luganda	Banana (as soft as bananas)	4
Unknown attribute				
	Machunde			1
	Kalimanzira			1
	Nakasugga			1

	Mid-north	Language	Meaning	Frequency
Area of origin/source	Ogwok	Langi	Introduced to place by Ogwok (name of person who introduced variety to the area)	4
Morphology	Icilcil	Langi	Beautiful (because it has beautiful leaves)	3
	Bao	Langi	Strong as timber (stems strong and tall)	5
Maturity period	Okonyoladak	Acholi	Konyo (help). Ladak (transferred to a new place). Best for new inhabitants because it matures early	2

	Southwest	Language	Meaning	Frequency
Area of origin/source	Bukalasa	Luganda	From a place called Bukalasa	7
Morphology	Kiteteyi	Runyankole	Canopy grows spreading out like a dress	1
	Bitamisi	Runyankole	No threads in tubers	2
	Nyakapimpiri	Runyankole	The shortest one (variety grows very short)	1
Taste	Rutuga	Runyankole	Strangling (can kill instantly if eaten fresh)	2
	Busukali	Runyankole	Sugar (it is as tasty as sugar)	1
Resilience	Kyebandira	Runyankole	To find your own way (it could grow anywhere planted	2
Yields	Kitengye	Runyankole	Piece of cloth (high yielding can be sold to buy clothes)	1
	Mpologoma	Luganda	Lion (variety grows large tubers)	1

	Eastern	Language	Meaning	Frequency
Ease to cook	Ofumbachai	Luganda	Boiling tea (cooks very fast)	4
	Ongada	not known	Not known	3
	Magana	Lugishu	Not known	5
Marketability	Mercury		It is as highly marketable as mercury	2
	Ekwataula	Ateso	Tail of a cow (marketable can earn money to buy a cow)	1
Taste	Jaribu	Kumam and swahili	Give it a try (taste then will know how tasty it is)	3
	Kwinini	Ateso	As bitter as quinine medicine	2
Yields	Emanyai	Ateso	Marriage (because its high yielding can feed enough people during a wedding	4
Maturity period	Omotoka	Ateso	Motor car. (quick like a car because it matures early)	4

	Mid-west			
	Variety	Language	Meaning	Frequency
*c) Categorization attribute*				
Area of origin/source	Kirimumpale	Runyoro	It is in the trouser (no idea about origin of meaning)	3
	Nyarale	Alur	Brought by Rale (name of person who introduced variety to the area)	3
	Bukalasa	Luganda	From a place called Bukalasa	10
	Timtim	Luo	Was introduced by Atim (name of person who introduced variety to the area)	11
	Kazimwenge	Swahiri	Grown for brewing	3
	Nyaraboke		Was first cultivated in a place called Laboke in Kiryandongo by Alur	21
	Nyaru-ucha	Alur	Obtained from Mr Ucha (name of person who introduced variety to the area)	3
	Nyaruzele	Alur	Introduced to community by Ozele (name of person who introduced variety to the area)	3
	Nyaeva	Rugungu	Brought to area by Eva (name of person who introduced variety to the area)	4
	Nyakakwa	Lugungu	Obtained from Kakwa people	4
	Rwakakaikuru	Runyoro	Old (been cultivated in the area for a very long time)	1
	Kalerenze	Runyoro	First cultivated by Kalerenze (name of person who introduced variety to the area)	1
	Nyakabibi	Rukonjo	Introduced by Kabibi (name of person who introduced variety to the area)	1
	Nyamururu	Runyoro	Cultivated by Alur people that live in Bunyoro and it is not eaten fresh	1
Maturity period				
	Nyakabiriti	Lugungu	Matchstick (matures early like lighting a match stick)	7
	Kabiriti enkooto	Runyoro	Big match box (matures fast like lighting a matchbox and tubers grow big)	6
	Agong	Alur	Lasts long (takes long to mature with a long storability in ground)	1
Taste				
	Karangwa	Runyoro	When eaten fresh one can die	6
	Kalinga	Lukonjo	Bitter	3
	Kigita	Lutooro	Looks and tastes like ghee	3
	Tongolo	Alur	Bitter variety	3
	Terengule	Alur	Very bitter	2
	Odieklewo	Acholi	Liked by goats	2
	Lyaholore	Runyoro	Taste the taste (because its tasty and soft)	6
	Ndiabulianoha	Runyoro	Could make very good flour mixed with millet variety called Ndiabulianoha	1
Morphology				
	Kabaho	Runyoro	Strong like timber	2
	Nylon		Very light like nylon	2
	Ekwiragura	Runyoro	Black (stems are dark)	3
	Rugogoma	Runyoro	Bending on the ground (mature stems bend and elongate horizontally)	4
	Nyalanda	Runyoro	Creeps (grows creeping)	3
	Kacumu	Runyoro	Spear (leaves are pointed like a spear)	1
	Katebe	Runyoro	Small. Variety as short as a small chair	1
	Kabundaire	Rukonjo	Grows short	1
	Kikofiira	Runyoro	Big hat (variety grows a canopy that looks like a big hat)	1
Ease to cook				
	Welobediyo	Alur	Welo (visitor) Bediyo (relax) meaning that visitor should not go hungry. The variety cooks very fast	3
	Nyakunyaku	Alur	Nyaku means a lady (liked by ladies because it cooks fast)	2
	Rwebitere	Runyoro	Dry cassava chips (variety makes good cassava flour)	1
	Mafuta	Runyoro	Oil (soft and easy to cook)	1
	Tonguda	Runyoro	So soft that time is not wasted during pounding	
Yield				
	Timpaigwamurwaire	Runyoro	Very high yielding that cannot be harvested by an ill person	2
	Nyasenge	Lugungu	To carry (high yielding may be heavy to carry by one person)	3
	Siba empali	Runyoro	Tighten your belt. Implies that it is high yielding, i.e., one has to tighten their belt to uproot.	2
	Kidimo	Runyoro	Hoe (related to early sprouting)	3
	Kirimumpali	Runyoro	In the trouser (high yielding one can sell and earn some money to keep in pocket)	1
	Majunza	Runyoro	Jiggers (produces many tubers compared to ’’eggs of a jigger’’)	1
	Andrua	Lugbara	Alone (saying ’’husband abandoned me (wife) let me grow this type of cassava to provide me food). Abandoned wife earns from it without support of husband	1
	Kalangwa butito	Lunyoro	Small short roots. Grows small short tubers	
Disease tolerance	Nyakhadhika	Lugbara	Other varieties can be affected by disease except this one	1
	Erwala bwangu	Runyoro	Susceptible to disease	1
Unknown attribute	Eriminiya	Lugbara	Not known	1
	Supiliya	Not known	Not known	1
	Omukolasi	Lukonjo	Not known	2
	Kanyari	Runyoro	Soot	2
	Kibalaya	Rukonjo	Not known	1
	Kasereghenye	Rukonjo	Not known	1
	Kachenche	Not known	Not known	1

### 3.4 Utilization of cassava local varieties

Primary purpose of cassava local varieties in study areas was home consumption and sale (76.2%). 23.3% responded that cassava local varieties are cultivated mainly for home consumption and 1.3% grow local varieties for sale only (Table 4).

**Table 4:** Utilization of cassava varieties in the study areas

Names of regions	For sale and home consumption (%)	For home consumption (%)	For sale only (%)	Total (%)
West	3.1	2.6	0.0	5.8
Central	11.5	9.9	0.5	21.5
Eastern	7.6	3.1	0.5	11.0
Northwest	17.8	0.3	0.3	18.3
Mid-west	32.5	6.8	0.0	39.3
Mid-north	3.7	0.5	0.0	4.2
Total	76.2	23.3	1.3	100.0

### 3.5 Farmers’ preferences for cultivation and selection of cassava local varieties

Generally the reasons reported for increased production included: high yields (165.1%), tastiness (144.20%), good cooking quality (103.90%), early maturity (76.20%), tolerance to disease (48.10%), storability (35.10%) and marketability (27.50%). In central Uganda, the highest ranked reason for increase in cultivation of varieties grown was taste (36%), in southwest it was early maturity period (34.6%), in eastern Uganda, northwest and mid-west, high yields were recorded highest at 34.60, 36.0 and 34.40%, respectively. In mid-north, however, taste and good cooking quality were the attributes most preferred by farmers at 29.40% in both categories (Table 5).

**Table 5:** Factors that farmers put into consideration when selecting cassava varieties in 6 agro-ecological zones of Uganda

	Region						
	Southwest (%)	Central (%)	Eastern (%)	Northwest (%)	Mid-west (%)	Mid-north (%)	Average (%)
Tastiness	21.10	36.00	23.10	14.00	20.60	29.40	24.0
High yielding	15.80	26.70	34.60	36.00	34.40	17.60	27.5
Early maturing	26.30	16.00	7.70	14.00	12.20	0.00	12.7
Tolerance to disease	10.50	10.70	3.80	6.00	5.30	11.80	8.0
Good cooking quality	21.10	2.70	19.20	20.00	11.50	29.40	17.3
Marketability	0.00	2.70	7.70	0.00	5.30	11.80	4.6
Storability in the ground	5.30	5.30	3.80	10.00	10.70	0.00	5.9

### 3.6 Reasons for decreased cultivation of cassava local varieties

Some farmers also believed that there was decreased cultivation of local cassava varieties due to reduced yields (211.3%), pests and disease susceptibility (67%), bitter taste (51.5%), lack of market (19.4%), introduction of improved varieties (220.50%), limited land for cultivation (19.70%) and long maturity period (10.6%). In southwestern and mid-western Uganda, farmers thought that the major reason for decreased production was reduced yields at 42.9 and 42.10%, respectively, while in central, mid-northern and northwestern Uganda, most farmers 35.3, 66.7 and 42.9%, respectively, believed that introduction of improved varieties contributed most to reduced cultivation of local varieties. In eastern Uganda, reduced yields and introduction of improved varieties at 35% were reported as the most contributing factors to decreased cultivation of cassava in the areas surveyed (Table 6).

**Table 6:** Farmer responses for decreased cultivation of cassava local varieties in 6 agro-ecological zones of Uganda

	Region						
	Southwest (%)	Central (%)	Eastern (%)	Northwest (%)	Mid-west (%)	Mid-north (%)	Average (%)
Reduced yields	42.90	29.40	35.00	28.60	42.10	33.30	35.22
Pests and disease susceptibility	0.00	17.60	30.00	7.10	12.30	0.00	11.17
Bitter taste	28.60	8.80	0.00	7.10	7.00	0.00	8.58
Poor market	0.00	8.80	0.00	3.60	7.00	0.00	3.23
Planting new improved varieties	14.30	35.30	35.00	42.90	26.30	66.70	36.75
Reduced land for cultivation	14.30	0.00	0.00	3.60	1.80	0.00	3.28
Long maturity period	0.00	0.00	0.00	7.10	3.50	0.00	1.77

### 3.7 Conservation practices undertaken by farmers and sources of planting material

Farmers reported that once a variety is introduced in the area for the first time they ensure that it is not lost if it possesses the traits of interest. This is achieved by replanting immediately after harvesting (331.10%), planting in multiple plots (171.70%), sharing with others in their networks to grow as backups (55.6%) and use of clean planting material (41.6%) (Table 7). Therefore, 385.4% reported that they use their own planting materials. But in case there is need to plant a specific variety that is not available on their farms, they can obtain it for free from other households within their social networks (161.2%). Occasionally stakes are obtained from outsiders for free (24.7%), are supplied by authorities (13.4%) and also purchased from other communities (15.6%) (Table 8).

**Table 7:** Methods of preserving cassava local varieties on farm

Responses	Regions						Total
	Southwest	Central	Eastern	Northwest	Mid-west	Mid-north	
Keep in multiple plots	42.90%	29.10%	43.30%	23.70	16.00%	16.70%	171.70%
Replanting	54.30%	67.50%	40.30%	39.60	51.60%	77.80%	331.10%
Share with others to grow as backup	0.00%	0.00%	11.90%	20.10%	18.00%	5.60%	55.6%
Use clean planting materials	2.90%	3.40%	4.50%	16.50%	14.30%	0.00%	41.6%
Total	35	117	67	139	244	18	620

**Table 8:** Sources of planting materials for cassava local varieties in the study area

Responses	Regions						Total
	West	Central	Eastern	Northwest	Mid-west	Mid-north	
Own	79.20%	68.20%	66.70%	51.90%	56.90%	62.50%	385.40%
Gift from person in community	16.70%	27.10%	26.70%	26.60%	32.80%	31.30%	161.2%
Gift from outside	0.00%	4.70%	6.70%	7.60%	5.70%	0.00%	24.7%
Supplied by authorities	0.00%	0.00%	0.00%	2.50%	4.60%	6.30%	13.4%
Purchased from outsider	4.20%	0.00%	0.00%	11.40%	0.00%	0.00%	15.6%
Total	24	85	45	79	174	16	423

## 4 Discussion

### 4.1 Cassava local varieties cultivated by farmers

Farmers are known to possess systems of categorizing and recognizing plant and animal species in their communities (Mtunguja et al. 2014). Similarly the naming of varieties as reported by farmers in the current study was based on the different attributes possessed by a variety, the origin of the variety (area where it was obtained) and the person who introduced the variety to the community and the morphological variation of the variety (Table 3). For instance, Welobediyo from mid-western Uganda means “relax,” farmers reported that the variety was named because it cooks very fast thus a saying that goes like “with Welobediyo, a visitor should relax since food will be available in the shortest time possible”. Sibampali from mid-western Uganda meaning “tighten your trouser” because variety is high yielding one should have enough energy to uproot it. Mercury variety named because farmers believe that it is as highly marketable as mercury. Gilgil variety from northwestern Uganda named after a village where it was mostly grown. Ocol meaning “dark” in Alur because leaves and stems of that variety are dark in color. Omoo meaning “coming together” in Lugbara, Clans that used to have conflicts were united after sharing Omoo variety. Katebe meaning “small chair” in Bantu language, because the variety grows short stems and branches thus the idiom “Mumpi nga Katebe translated, as short as a chair”. The naming of cassava varieties as reported by farmers in this study is similar to what has been reported in other regions and crop varieties. For instance, Mehta et al. (2009) reported that the naming criteria of rice varieties in the Himalayas is based on morphological traits, environmental adaptability, agronomic traits, place of origin and local recipes. In Tanzania, however, Mtunguja et al. (2014) observed that famers use mainly well adapted morphological descriptors to distinguish between cassava varieties. They could successfully distinguish between bitter and sweet varieties.

In Africa, some communities breed local crop varieties on the basis of indigenous knowledge and use local taxonomy in selecting and naming varieties (Haugerud and Collinson 1990; Almekinders et al. 1994). In mid-west agro-ecological zone specifically in the district of Buliisa, some communities reported a practice of exploring cassava attributes by germinating cassava seeds and evaluating the seedling specifically for culinary attributes, disease susceptibility and yield. In this region, some varieties are named according to the individual who sprouted, evaluated and later distributed them to the entire community. Varieties such as Nyarale “introduced by Rale”, Nyaru-ucha “introduced by Ucha”, Nyaruzele “introduced by Zele” and Nyaeva “introduced by Eva” were generated from such practices. This raises the importance of local breeders within communities and the importance of establishing community-based breeding programs for cassava. The study has shown that, farmers involved in local breeding are highly appreciated to an extent that the varieties generated after such practices are named after them. Therefore, farmers’ involvement in planning and execution of breeding strategies could enhance adoption rates of improved varieties.

Occasionally, cassava seedlings randomly germinate and grow among vegetatively propagated plants. However, due to the highly heterozygous nature of cassava it is most likely that the seeds are genetically dissimilar from the parents. And it’s also possible that the seedlings are disease free since vegetatively propagated material serve as the major source of diseases (Elias et al. 2000). Farmers normally get interested in these seedlings, evaluate and protect them for the next growing season if they possess agronomic and culinary values. In this way, farmers incorporate genetic variability from sexual reproduction into the local cassava variety gene pool.

### 4.2 Utilization of cassava varieties

The study indicated that most farmers 76.2% cultivate cassava local varieties for both home consumption and sale. With this category being dominated by farmers from mid-west (32.5%), northwest (17.8%) and central Uganda (11.5%), respectively (Table 4), in mid-western Uganda farmers with small acreage of land grow cassava mostly for home consumption. Farmers from central Uganda where Banana is a major food crop also commonly grow cassava for home consumption, consumed fresh as snacks and making flour for pancakes for sale. During the survey it was noted that commercial farmers mostly grow improved varieties that are known to be high yielding with early maturity period and do not care much about selection and on-farm retention or conservation since improved varieties are distributed from government and non-government organizations (NGO) periodically. The growing of local varieties mostly for home consumption has ensured on-farm conservation, since farmers always keep a few but a variety of plants for food security and also to complement the positive and negative traits that could be in the local varieties. Similar to previous findings from other regions, in the current study most farmers believed that cassava local varieties are better for food security than improved varieties because they have a longer storability in the soil. Bitter varieties make good flour for food and local beer and therefore helpful during drought and also thought to be less susceptible to disease (Mtunguja et al. 2014; Akintunde and Obayelu 2016). Bitterness is also thought to be a food security measure since it is avoided by thieves. These findings emphasize the need to incorporate farmer preferences in conservation and breeding strategies in order to improve productivity and sustainable use of cassava genetic resources.

### 4.3 Production dynamics of cassava local varieties

The farmers that participated in the study had been cultivating cassava for not less than 5 years up to 50 years and more. They were therefore believed to be knowledgeable about cassava production dynamics over the years. Most farmers believed that cultivation of cassava local varieties has increased in the past few years (Table 5). The major reasons for increased cassava production in general included: tastiness (144.20%), high yields (165.1%), early maturity (76.10%), disease tolerance (48.10%), good cooking quality (103.9%), marketability (27.5%) and storability (35.10%). Farmers emphasized culinary attributes (taste), high yield and cooking quality as the major reason for variety cultivation and retention. Elias et al. (2000) observed that while farmers generally prefer high yielding varieties, they may preserve lower yielding varieties in parallel with more productive varieties, due to cultural preferences such as taste or cooking quality. In the process, farmers manage the risk of a calamitous crop loss by keeping several different varieties in production at the same time and often in the same field. In northwestern, mid-northern and some parts of eastern Uganda, cassava is mostly processed prior to consumption due to the high cyanogenic compounds in the cultivated varieties (Balyejusa Kizito et al. 2006). In these regions, bitter varieties are considered tastier after processing than the sweet varieties and thus high starch content is a key trait that farmers look for in the varieties cultivated.

### 4.4 Conservation practices undertaken by farmers on the cassava varieties

It is not unusual for farmers to exchange stem cuttings with their neighbors and neighboring communities, resulting in fields with a mixture of local cassava varieties (Andersson and de Vicente 2010). And it is not common that cassava planting materials for local varieties are sold or bought. Although farmers may not deliberately conserve cassava genetic resources, they do so through traditional practices that ensure on-farm retention of varieties over years.

Cassava farmers in Uganda use various practices to ensure retention of varieties on farm. They plant varieties in multiple plots (40.3%), replant immediately after harvesting (84.2%), share with others (21.1%) and use clean planting material (17.4. %). In case planting for some reason does not happen immediately after harvesting, farmers reported that stakes can be kept viable for some time under the shade preferably in an upright position until the next planting. These methods ensure that in case of any catastrophic events, varieties are not lost. With the prevalence of viral diseases, some farmers have learnt to recognize symptoms associated with cassava viral diseases and select planting material from plants that are asymptomatic (Table 7). This strategy not only ensures availability of food and seed for the next planting but also reduces the spread of diseases on farm and within the community. The farmers were commended and encouraged to continue planting disease-free cuttings in order to reduce the spread of diseases.

The information generated during this study indicates that farmers in most areas where cassava is grown have more or less similar practices of maintenance and preservation of cassava genetic resources (Balyejusa Kizito et al. 2006; Elias et al. 2001; Mtunguja et al. 2014; Akintunde and Obayelu 2016). However, it was observed during this study that farmers from the study areas are not aware of the scientific importance of conservation of cassava genetic resources rather preserve varieties for the sake of supporting their households’ needs. Therefore, government and development partners should invest in sensitizing farmers about the importance of on-farm conservation of crop genetic resources for future use.

## 5 Conclusion

The information obtained from this study indicates that farmers are still growing cassava local varieties despite the distribution of disease-tolerant and high yielding improved varieties. It is also clear that farmers have preferences that influence the decisions taken to retain or abandon particular varieties. Based on their local knowledge, farmers understand the need to grow a diversity of cassava as this can help in times of scarcity. Although improved varieties have enabled continued cassava production in Uganda and the region, their cultivation and adoption may reduce the number of varieties cultivated by farmers leading to erosion of genetic diversity (Tripp 1996). As the government of Uganda promotes research toward cassava improvement, measures should be put in place to ensure protection and sustainable utilization of its genetic resources.

## Notes

**Open Access** This article is distributed under the terms of the Creative Commons Attribution 4.0 International License (http://creativecommons.org/licenses/by/4.0/), which permits unrestricted use, distribution, and reproduction in any medium, provided you give appropriate credit to the original author(s) and the source, provide a link to the Creative Commons license, and indicate if changes were made.

## References

[cit0001] Akintunde O. O., & Obayelu O. A. (2016). Farmers’ perception of on-farm conservation of cassava biodiversity in Ogun State, Nigeria. International Food Research Journal, 23(5), 2265–2270.

[cit0002] Alicai T., Omongo C., & Maruthi M. (2007). Re-emergence of cassava brown streak disease in Uganda. Plant Disease, 91, 24–29. 30781061 10.1094/PD-91-0024

[cit0003] Almekinders C. J. M., Louwaars N. P., & de Bruijn G. H. (1994). Local seed systems and their importance for an improved seed supply in developing countries. Euphytica, 78, 207–216.

[cit0004] Andersson M. S., & de Vicente M. C. (2010). Cassava, manioc, yuca In Andersson M. S. & de Vicente M. S. (Eds.), Gene flow between crops and their wild relatives (pp. 125–146). Baltimore: Johns Hopkins University Press.

[cit0005] Balyejusa Kizito E. (2006). Genetic and root growth studies in cassava (*Manihot esculenta Crantz*): Implications for breeding. Doctor’s dissertation. ISSN 1652-6880, ISBN 91-567-7131-1.

[cit0006] Balyejusa Kizito E., Chiwona-Karltun L., Egwang T., Fregene M., & Westerbergh A. (2007). Genetic diversity and variety composition of cassava on small scale farms in Uganda: An interdisciplinary study using genetic markers and farmer interviews. Genetica, 130, 301–318. 17082904 10.1007/s10709-006-9107-4

[cit0007] Balyejusa Kizito E., Rönnberg-Wästljung A.-C., Egwang T., Gullberg U., Fregene M., & Westerbergh A. (2006). Quantitative trait loci controlling cyanogenic glucoside and dry matter content in cassava (*Manihot esculenta Crantz*) roots. Hereditas, 144(4), 129–136. 10.1111/j.2007.0018-0661.01975.x 17850597

[cit0008] Elias M., McKey D., Panaud O., Anstett M. C., & Thierry R. (2001). Traditional management of cassava morphological and genetic diversity by the Makushi Amerindians (Guyana, South America): Perspectives for on-farm conservation of crop genetic resources. Euphytica, 120, 143–157.

[cit0009] Elias M., Panaud O., & Robert T. (2000). Assessment of genetic variability in a traditional cassava (Manihot esculenta Crantz) farming system, using AFLP markers. Heredity, 85, 219–230. 11012725 10.1046/j.1365-2540.2000.00749.x

[cit0010] FAO (2009). FAO and traditional knowledge: The linkages with sustainability, food security and climate change impacts (pp. 184–185). Rome: FAO.

[cit0011] FAO (2010). The second report on the state of the world’s plant genetic resources for food and agriculture. Rome.

[cit0012] Gwali S., Okullo J. B. L., Eilu G., Nakabonge G., Nyeko P., & Vuzi P. (2011). Traditional management and conservation of Shea trees (*Vitellaria paradoxa* subspecies nilotica) in Uganda. Environment, Development and Sustainability, 14(3), 347–363.

[cit0013] Haugerud A., & Collinson M. P. (1990). Plants, genes and people: Improving of plant breeding in Africa. Experimental Agriculture, 26(3), 341–362.

[cit0014] Hillocks R. J. (2002). Cassava in Africa In Hillocks R. J., Thresh J. M. & Bellotti A. C. (Eds.), Cassava: Biology, production, and utilization (pp. 41–54). Wallingford: CAB International.

[cit0015] Jansson C., Westerbergh A., Zhang J., Hu X., & Sun C. (2009). Cassava, a potential biofuel crop in (the) People’s Republic of China. Applied Energy, 86, 95–99.

[cit0016] Kawuki R. S., Kaweesi T., Esuma E., Pariyo A., Kayondo S. I., Ozimati A., et al. (2016). Eleven years of breeding efforts to combat cassava brown streak disease. Breeding Science Preview, 66(4), 560–571. 10.1270/jsbbs.16005 PMC5010303 27795681

[cit0017] Langlands J. (1972). Cassava in Uganda. Uganda Journal, 10, 273–286.

[cit0018] Legg J. P. (1999). Emergence, spread and strategies for controlling the pandemic cassava mosaic virus disease in East and Central Africa. Crop Protection, 18, 627–637.

[cit0019] Legg J. P., & Fauquet C. M. (2004). Cassava mosaic geminiviruses in Africa. Plant Molecular Biology, 56(4), 585–599. 15630622 10.1007/s11103-004-1651-7

[cit0020] Mehta P. S., Rathi R. S., Negi K. S., & Ojha S. N. (2009). Farmer’s criteria for naming crop varieties: A case on rice varieties in Kumaon Himalaya of Uttarakhand. Indian Journal of Plant Genetic Resources, 22(3), 215–220.

[cit0021] Mtunguja M. K., Laswai H. S., Muzanila Y. C., & Ndunguru J. (2014). Farmer’s knowledge on selection and conservation of cassava (*Manihot esculenta*) genetic resources in Tanzania. Journal of Biology, Agriculture and Healthcare, 4(10), 122–129.

[cit0022] Nassar N. M. A. (2000). Cytogenetics and evolution of cassava (Manihot esculenta Crantz). Genetics and Molecular Biology, 23, 1003–1014.

[cit0023] Nassar N. M. A., & Ortiz R. (2006). Cassava improvement: Challenges and impacts. Journal of Agricultural Science, 145, 163–171.

[cit0024] Negi S. C. (2010). Traditional knowledge and biodiversity conservation: Examples from Uttarakhand, Central Himalaya. Mountain Research and Development, 30(3), 259–265.

[cit0025] Ng N. Q., & Ng S. Y. C. (2002). Genetic resources and conservation In Hillocks R. J., Thresh J. M. & Bellotti A. C. (Eds.), Cassava: Biology, production, and utilization (pp. 167–177). Wallingford: CABI Publishing.

[cit0026] Nuwamanya E., Bagumal Y., Kawuki R. S., & Rubaihayo P. R. (2009). Quantification of starch physicochemical characteristics in a cassava segregating population. African Crop Science Journal, 16, 191–202.

[cit0027] OECD (2014). Environment directorate Joint meeting of the chemicals committee and the working party on chemicals, pesticides and biotechnology. Consensus document on the biology of cassava (Manihot esculenta Crantz). Series on Harmonisation of Regulatory Oversight in Biotechnology, No. 57, OECD, Paris Available on the BioTrack website at (http://www.oecd.org/chemicalsafety/).

[cit0028] Okogbenin E., Porto M. C. M., Egesi C., Mba C., Ospinosa E., Santos L. G., et al. (2007). Marker aided introgression of CMD resistance in Latin American germplasm for genetic improvement of cassava in Africa. Crop Science, 47, 1895–1904.

[cit0029] Parajuli D. R., & Das T. (2013). Indigenous knowledge and biodiversity: Interconnectedness for sustainable development. International Journal of Scientific & Technology Research, 2(8), 220–224.

[cit0030] Peroni N., & Hanazaki N. (2002). Current and lost diversity of cultivated varieties, especially cassava, under swidden cultivation systems in the Brazilian Atlantic forest. Agriculture, Ecosystems & Environment, 92, 171–183.

[cit0031] Tripp R. (1996). Biodiversity and modern crop varieties: Sharpening the debate. Agriculture and Human Values, 13(4), 48–63.

[cit0032] Turyagyenda L. F., Kizito E. B., Ferguson M. E., Baguma Y., Harvey J. W., Gibson P., et al. (2012).Genetic diversity among farmer preferred cassava landraces in Uganda. African Crop Science Journal, 20, 15–30.

[cit0033] UBOS (2010). Crop census and production report In: Uganda census of Agriculture. Uganda Bureau of Statistics, Ministry of Agriculture, Animal Industry and Fisheries, Entebbe.

[cit0034] Wilder B. T., O’Meara C., Monti L., & Nabhan G. P. (2016). The importance of indigenous knowledge in curbing the loss of language and biodiversity. BioScience, 66(6), 499–509.

